# Histamine deficiency exacerbates myocardial injury in acute myocardial infarction through impaired macrophage infiltration and increased cardiomyocyte apoptosis

**DOI:** 10.1038/srep13131

**Published:** 2015-08-17

**Authors:** Long Deng, Tao Hong, Jinyi Lin, Suling Ding, Zheyong Huang, Jinmiao Chen, Jianguo Jia, Yunzeng Zou, Timothy C. Wang, Xiangdong Yang, Junbo Ge

**Affiliations:** 1 Shanghai Institute of Cardiovascular Diseases, Zhongshan Hospital, Fudan University, Shanghai, 200032, China; 2 Department of Cardiac Surgery, Zhongshan Hospital, Fudan University, Shanghai, 200032, China; 3 Institutes of Biomedical Sciences, Fudan University, Shanghai, China; 4 Department of Medicine and Irving Cancer Research Center, Columbia University, New York, NY 10032, USA

## Abstract

Histamine is a biogenic amine that is widely distributed and has multiple functions, but the role it plays in acute myocardial infarction (AMI) remains unclear. In this study, we investigated the origin and contribution of endogenous histamine to AMI. Histidine decarboxylase (HDC) is the unique enzyme responsible for histamine generation. Using HDC-EGFP bacterial artificial chromosome (BAC) transgenic mice in which EGFP expression is controlled by the HDC promoter, we identified HDC expression primarily in CD11b^+^Gr-1^+^ immature myeloid cells (IMCs) that markedly increase in the early stages of AMI. Deficiency of histamine in HDC knockout mice (HDC^−/−^) reduced cardiac function and exacerbated the injury of infarcted heart. Furthermore, administering either an H_1_ receptor antagonist (pyrilamine) or an H_2_ receptor antagonist (cimetidine) demonstrated a protective effect of histamine against myocardial injury. The results of *in vivo* and *in vitro* assays showed that histamine deficiency promotes the apoptosis of cardiomyocytes and inhibits macrophage infiltration. In conclusion, CD11b^+^Gr-1^+^ IMCs are the predominant HDC-expressing sites in AMI, and histamine plays a protective role in the process of AMI through inhibition of cardiomyocyte apoptosis and facilitation of macrophage infiltration.

Myocardial infarction (MI) involves multiple pathological processes that are initiated by cardiomyocyte necrosis, followed by leukocytes recruitment, formation of granulation tissue, and myocardial remodeling. Acute inflammation has proved to play an essential role in the removal of dead cardiomyocytes and the subsequent healing process^1^,^2^. Numerous immune cells and cytokines have been demonstrated to participate in the process of MI, and to maintain the inflammation at a balanced and orchestrated level^3^. A much too aggressive or attenuated inflammatory response could affect myocardial function and the healing process.

Histamine is a biogenic amine which is widely distributed and has many functions^4^,^5^,^6^,^7^. Several studies have found that the levels of histamine in the serum and myocardium increased significantly in acute myocardial infarction (AMI)^8^,^9^,^10^,^11^,^12^. Mast cells have been considered to be the primary cellular source of histamine. However, previous studies have also indicated that the number of mast cells in the infarcted heart does not increase dramatically in the early stage of AMI^13^,^14^,^15^. Some studies showed immediately increased degranulation of mast cells following myocardial ischemia^16^,^17^,^18^, but it seems that this reaction is not long-lasting^19^. These results suggested that, in the early stage of AMI, mast cells might not be the predominant source of increased histamine. In the cardiovascular system, histamine is documented to be vasoactive, inotropic, chronotropic, and arrhythmogenic^20^,^21^,^22^. However, a role for histamine in the pathogenesis of specific cardiovascular diseases remains controversial. Several studies suggested that histamine could be a negative factor in atherosclerosis (AS) and vascular remodeling, but few studies have directly explored the role of histamine in AMI^10^,^13^,^23^,^24^,^25^.

Histidine decarboxylase (HDC) is the unique enzyme that converts L-histidine to histamine. In a recent study using HDC-EGFP transgenic reporter mice^26^, bone marrow and spleen derived CD11b^+^Gr-1^+^ immature myeloid cells (IMCs) were identified as the primary HDC-expressing site. Given that many immune cells are mobilized into the circulation and infarcted myocardium during the process of AMI, we hypothesized that HDC^+^ IMCs might be recruited to the cardiovascular system and contribute to histamine production, and thus play an important role in AMI. In the present study, HDC-EGFP transgenic reporter mice and histamine deficiency mice (HDC knockout, HDC^−/−^) were introduced to clarify the expression and function of HDC and histamine in AMI.

## Results

### The expression of histamine increases significantly after AMI

**Figure 1:**
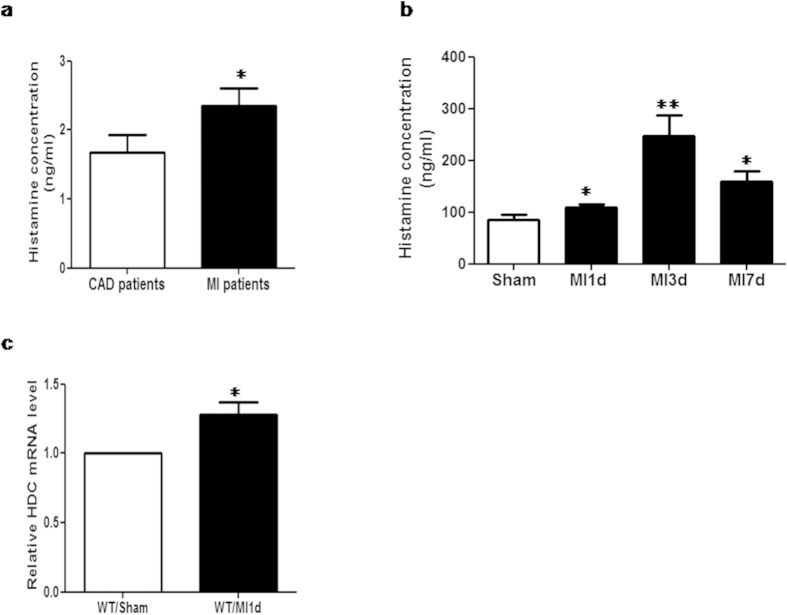
The level of histamine increases after AMI. (**a**) In patients with AMI (within 24 hrs from onset), the level of histamine in the serum increased significantly compared to that in controls group (patients with coronary artery diseases, not MI) (MI: 2.35 ± 0.96 ng/ml vs Controls: 1.68 ± 0.7 ng/ml, *p < 0.05; n = 14 in MI group, n = 10 in controls group). (**b**) MI was induced by surgery in the murine model. MI mice had higher level of histamine in the serum than the sham group (Sham: 86.2 ± 11.71 ng/ml, MI1d: 110 ± 5.07 ng/ml, MI3d: 246.4 ± 40.42 ng/ml, MI7d: 160.1±19.01 ng/ml, *p < 0.05 vs sham, **p < 0.01 vs sham; n = 5–8). (**c**) HDC mRNA level in the blood cells of mice increased after MI (*p < 0.05 vs sham; n = 3).

To investigate the expression of histamine during the process of AMI, we analyzed the levels of histamine in the serum of patients and a mouse model with AMI by ELISA. First, serum samples were collected from 14 patients with angiographically confirmed MI (2.5 hours to 15 hours from onset) and from 10 control patients with coronary artery diseases (not MI) confirmed by angiography. The results showed that the levels of histamine increased significantly in the peripheral blood of MI patients compared to that in controls (Fig. 1a). Furthermore, a murine model of AMI was established to examine the histamine concentration in the serum at 1 day, 3days, and 7days post MI induction, respectively. Similar to our findings in patients, results from the mouse model indicated that the levels of histamine were also elevated after MI in mice (Fig. 1b), with the peak value (MI 3d: 246.4 ng/ml vs Sham: 86.2 ng/ml, **p < 0.01) appearing at 3 days post MI. In addition, quantitative-RT-PCR results confirmed that the expression of HDC mRNA increased in the blood mononuclear cells of MI mice, which suggests the elevation of histamine levels is related to increased expression of HDC gene (Fig. 1c). Taken together, these results demonstrate that there are increases of histamine and HDC expression in the circulation following myocardial infarction in both patients and a murine model. However, the source and role of histamine in the process of MI remains largely unclear.

### CD11b^+^Gr-1^+^ immature cells are the predominant HDC-expressing sites during AMI

**Figure 2:**
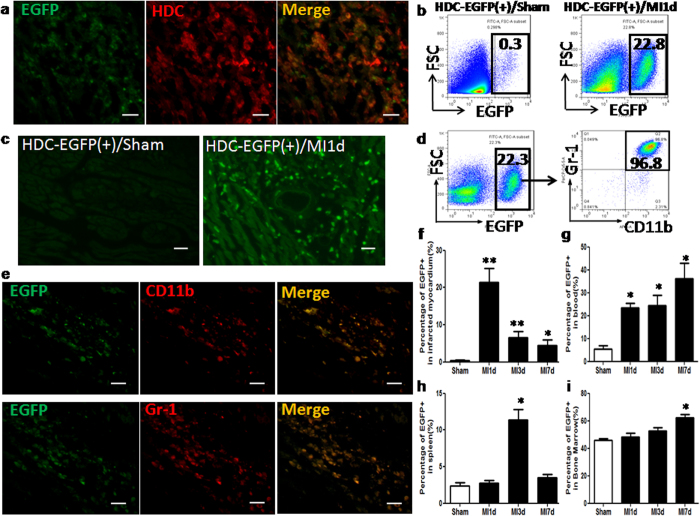
Identification of the cellular source of histamine during AMI. AMI model was established in HDC-EGFP mice. (**a**) Immunofluorescence staining confirmed the expression of HDC in EGFP^+^ cells (scale bar = 20 μm). (**b**) At 1d post MI, FACS analysis of the increasing percentage of EGFP^+^ cells in the infarcted myocardium (Sham: 0.39 ± 0.13% vs MI: 21.37 ± 3.8%, p<0.01; n = 5). (**c**) Representative images of hearts in HDC-EGFP^+^ mice with MI1d compared with sham, showing a large amount of EGFP^+^ cells infiltration (scale bar = 20 μm). (**d**) FACS analysis characterized EGFP^+^ cells isolated from the infarcted heart with myeloid cell markers: anti-CD11b and anti-Gr-1. (**e**) Immunofluorescence staining showed most of EGFP^+^ cells (above 90%) were CD11b^+^ and Gr-1^+^ immature myeloid cells (scale bar = 20 μm). (**f**–**i**) The expression patterns of EGFP^+^ cells over time in the infarcted myocardium examined by FACS. (**f**) in the myocardium, (**g**) in the blood, (**h**) in the spleen, (**i**) in the bone marrow are shown (**p < 0.01 vs Sham, *p < 0.05 vs Sham; n = 5–8).

Given that previous studies demonstrated that histidine decarboxylase (HDC) is the unique enzyme that converts L-histidine to histamine, we sought to identify the HDC-expressing cells in MI mice that likely serve as the cellular source of histamine in the cardiovascular system. Immunofluorescence studies were carried out on HDC-EGFP bacterial artificial chromosome (BAC) transgenic reporter mice (HDC-EGFP), and confirmed the expression of HDC in EGFP^+^ cells located in the infarcted heart with anti-HDC staining (Fig. 2a). At 1 day after MI, FACS (fluorescence activated cell sorter) and immunofluorescence data showed that EGFP^+^ cells increased dramatically in the infarcted myocardium (Fig. 2b,c). Furthermore, FACS and immunofluorescence data also demonstrated that approximate 95% of EGFP^+^ cells in the infarcted heart are CD11b^+^Gr-1^+^ immature myeloid cells (IMCs) (Fig. 2d,e). In addition, FACS data revealed the changing patterns of EGFP^+^ cells over time in the infarcted myocardium, blood, spleen, and bone marrow (Fig. 2f–i), with an early peak (day 1) of EGFP^+^ cells in the infarcted myocardium and later peaks in the spleen (day 3), blood (day 7), and bone marrow (day 7). Thus, the current data demonstrate that HDC-expressing CD11b^+^Gr-1^+^ IMCs are the major cellular source of histamine increase in the serum and infarcted heart of MI mice.

### Histamine deficiency exacerbates the injury of infarcted heart

**Figure 3:**
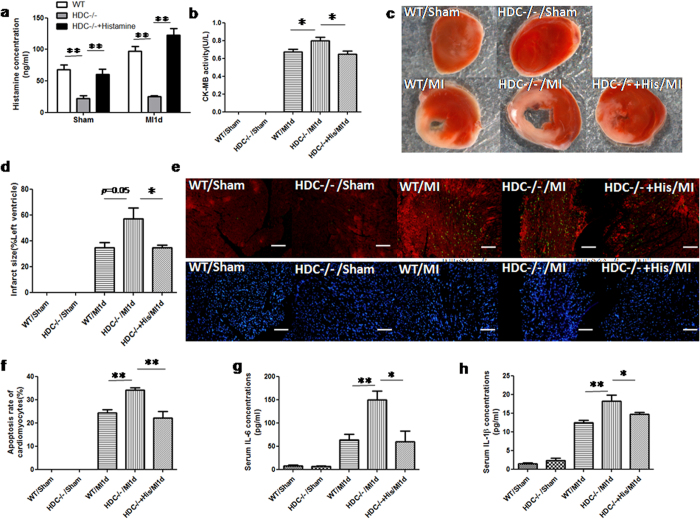
Histamine deficiency exacerbates the injury of infarcted heart. (**a**) ELISA assays of serum histamine confirmed HDC^−/−^ mice are deficient in histamine (**p < 0.01 vs WT; n = 4–6). (**b**) 1d post MI, higher CK-MB activity was examined in the serum of HDC^−/−^ mice (*p < 0.05 vs WT; n = 5), which could be abrogated by exogenous histamine administration (*p < 0.05 vs HDC^−/−^; n = 5). (**c,d**) Representative mid-myocardial cross-sections of TTC-stained hearts. Larger infarct size in HDC^−/−^ mice was demonstrated(*p = *0.05 vs WT; n = 4), which could also be abrogated by exogenous histamine administration (*p < 0.05 vs HDC^−/−^; n = 4–5). (**e,f**) TUNEL and anti-α-actinin staining identified apoptosis of cardiomyocytes of MI mice. Nuclei were displayed by DAPI staining. Representative images from WT, HDC^−/−^ and HDC^−/−^ +Histamine infarcts 1d post MI are shown (scale bar = 50 μm). Histamine deficiency increased the apoptosis of cardiomyocytes (**p < 0.01 vs WT; n = 4), but was abrogated by exogenous histamine injection (**p < 0.01 vs HDC^−/−^; n = 4). (**g**,**h)** 1d post MI, HDC^−/−^ mice had higher levels of IL-6(**g**) and IL-1β(**h**) in the serum, compared to that in WT counterparts(**p < 0.01 vs WT; n = 5–6).

To investigate the role of histamine in the pathogenesis of MI, histamine-deficient mice (HDC knockout, HDC^−/−^) were used to establish a MI model using a standard vascular ligation. First, ELISA assays confirmed lower levels of serum histamine in HDC^−/−^mice compared with wild type (WT) mice (Fig. 3a). The limited concentration of histamine in HDC^−/−^ mice is likely due to histamine uptake from a standard murine diet. Furthermore, we observed that CK-MB (creatine kinase-MB) activity increased in the serum of HDC^−/−^ mice compared with WT mice 1 day post MI (Fig. 3b). The results of triphenyltetrazolium chloride (TTC) staining demonstrated larger infarct size in HDC^−/−^ mice compared to WT controls (Fig. 3c,d). To evaluate the effects of histamine on the left ventricular function of MI mice, HDC^−/−^ and WT mice with MI were evaluated with two-dimensional, high-resolution echocardiography 7days post MI. HDC^−/−^ mice displayed a significantly reduced left ventricular ejection fraction (LVEF), compared with the WT mice (Table 1). In addition, TUNEL studies showed that histamine deficiency increased the number of apoptotic α-actinin^+^ cardiomyocytes in HDC^−/−^ mice compared with WT mice 1 day after MI (Fig. 3e,f). To confirm the protective function of histamine in the infarcted heart, exogenous histamine was administrated in HDC^−/−^ mice before ligation. Intraperitoneal injection of histamine at the dose of 4 mg/kg/d for 3 consecutive days pre-MI increases the levels of histamine in these HDC^−/−^mice, which approached that in WT mice (Fig. 3a). In the histamine-treated HDC^−/−^ mice, the level of CK-MB activity and infarct size were reduced, LVEF value was increased and the number of apoptotic cardiomyocytes decreased (Fig. 3b–f). In addition, ELISA assay revealed that the expression of inflammatory cytokines interleukin 6 (IL-6) and interleukin 1-beta (IL-1β) increased significantly in the serum of HDC^−/−^ mice compared with WT and histamine-treated HDC^−/−^ groups (Fig. 3g,h). Taken together, these results suggest a protective role of HDC and endogenous histamine in mitigating myocardial injury following AMI.

### IMCs derived histamine inhibits the apoptosis of cardiomyocytes

**Figure 4:**
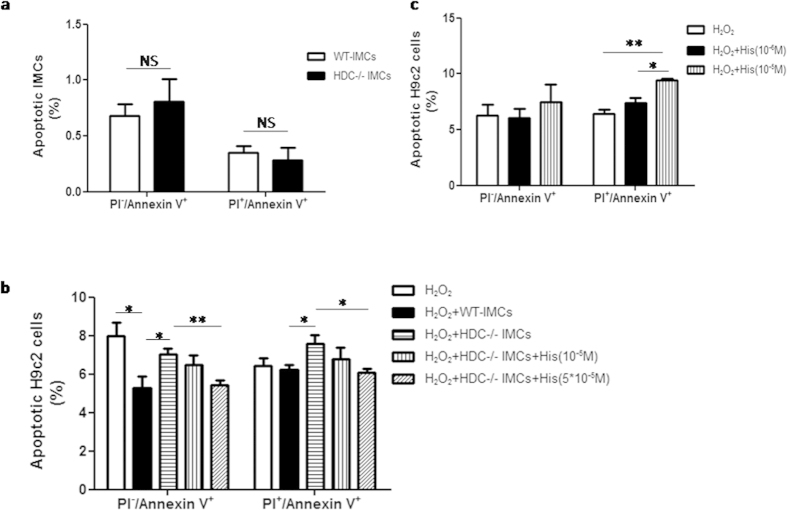
IMCs derived histamine protects against the apoptosis of cardiomyocytes. H9c2 cells were co-cultured with CD11b^+^Gr-1^+^ IMCs isolated from HDC^−/−^ or WT mice and H_2_O_2_ (100 μmol/L) was added to induce oxidative stress. After 48 hrs, Annexin V-PI staining was performed to detect apoptosis, H9c2 cells were distinguished from IMCs by anti-CD11b. (**a**) FACS data showed IMCs from both groups were equally sensitive to H_2_O_2_ (n = 4). (**b**) FACS analysis showed the increases of both early and late apoptosis of H9c2 cells co-cultured with HDC^−/−^ IMCs (*p < 0.05 vs WT; n = 4). These effects could be abrogated by exogenous addition of histamine (5*10^−5^M) (*p < 0.05 vs HDC^−/−^, **p < 0.01 vs HDC^−/−^; n = 4). (**c**), Exogenous addition of histamine alone could increase the apoptosis of H9c2 cells (**p < 0.01 vs control; n = 3).

To further clarify the effect of IMC-derived histamine on cardiomyocyte apoptosis, we performed an *in vitro* experiment with H9c2 rat myocardioblasts and CD11b^+^Gr-1^+^ IMCs isolated directly from the bone marrow of HDC^−/−^and WT mice. IMCs were co-cultured with H9c2 cells for 48 hours and H_2_O_2_ (100 μmol/L) was added to mimic a microenvironment with oxidative stress. We found that the percentage of apoptotic AnnexinV^+^ IMCs was not significantly changed in response to oxidative stress in single IMCs culture system isolated from both HDC^−/−^ and WT mice (Fig. 4a). However, using H9c2 cells co-cultured with bone marrow-derived IMCs, FACS data demonstrated that histamine deficiency increased the percentage of apoptotic H9c2 cells (AnnexinV^+^PI^−^ marks early stage apoptotic cells and AnnexinV^+^PI^+^ marks later stage apoptotic cells, Fig. 4b). Enhanced apoptosis in H9c2 cells co-cultured with HDC^−/−^ mice-derived IMCs could be abrogated by administration of exogenous histamine (Fig. 4b). To determine whether histamine alone has a protective effect on cardiomyocytes, H9c2 cells were treated with different concentrations of histamine. FACS data showed that low concentrations of histamine (10^−6 ^M) had no effect on the apoptosis of H9c2 cells, whereas higher dose of histamine (10^−5 ^M) actually promoted the apoptosis of H9c2 cells (Fig. 4c). Taken together, these data suggest that histamine exerts its anti-apoptotic effect indirectly, partially through regulating the functions of IMCs in the setting of oxidative stress, rather than through direct effects on cardiomyocytes.

### Histamine deficiency impairs macrophage infiltration and suppresses the healing process

**Figure 5:**
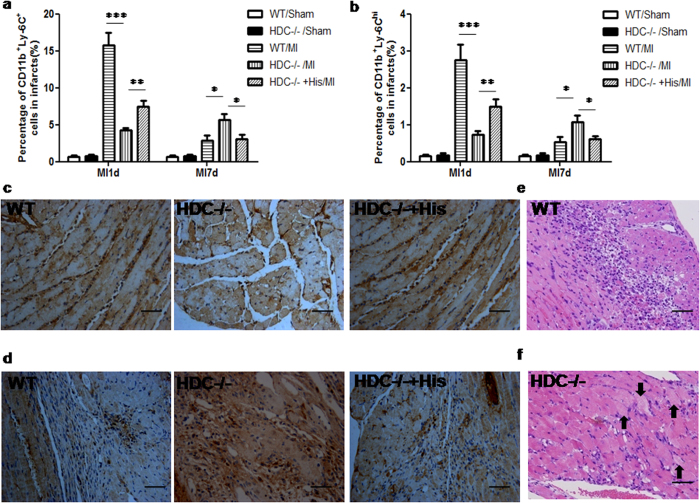
Histamine deficiency impairs macrophage infiltration and suppresses the healing process. (**a**) 1d post MI, FACS data showed that HDC^−/−^ mice had a significantly lower percentage of CD11b^+^Ly6C^+^ total macrophages in the infarcted heart than the WT counterparts (WT: 15.72 ± 1.72% vs HDC^−/−^:4.22 ± 0.39%, ***p < 0.001; n = 10–12). 7d post MI, the percentage of macrophages in the infarcted heart of WT mice declined, but in HDC^−/−^ mice it still went up and even exceeded that of WT mice (*p < 0.05 vs WT; n = 5–7). These effects could be abrogated by exogenous histamine (His) injection (**p < 0.01 vs HDC^−/−^, *p < 0.05 vs HDC^−/−^; n = 6–9). (**b**) As for CD11b^+^Ly6C^high^ M1 macrophages, the similar trend was observed. (**c,d)** Representative images of anti-CD68 immunohistochemistry study in the hearts 1d ((**c**) scale bar = 50 μm) and 7d ((**d**) scale bar = 50 μm) post MI. The trends were consistence with that in FACS data. (**e,f**) 3d post MI, dead cardiomyocytes were almost completely replaced by granulation tissue in WT mice ((**e**), HE staining, scale bar = 50 μm). In contrast, at the same time point, HDC^−/−^ mice showed incomplete granulation tissue formation and persistent presence of injured cardiomyocytes in the infarct ((**f**), HE staining, scale bar = 50 μm).

Given that macrophages are essential for the healing process at the earliest stages of MI^27^,^28^,^29^, and histamine exhibits a protective effect on the process of AMI, we hypothesized that histamine can promote macrophage infiltration that contributes to myocardial healing. At 1 day after MI, FACS data showed that HDC^−/−^ mice had a significantly lower number of total CD11b^+^Ly6C^+^ macrophages (Fig. 5a) and CD11b^+^Ly6C^high^ M1-type macrophages (Fig. 5b) in the infarcted heart, compared to WT counterparts. At 7 days after MI, the percentage of CD11b^+^Ly6C^high^ M1-type macrophages in the infarcts of WT mice declined dramatically, while it continued to increase in HDC^−/−^ mice, and even exceeded that of WT mice (Fig. 5b). All of these effects in HDC^−/−^ MI mice could be abrogated by exogenous histamine injection (Fig. 5a,b). The results of immunohistochemistry staining with anti-CD68 demonstrated a similar trend of macrophage infiltration as that revealed by FACS (Fig. 5c,d). In WT mice, early leukocytes infiltration was followed by debridement of the necrotic tissue, resulting in almost complete replacement of dead cardiomyocytes with granulation tissue 3 days after MI (Fig. 5e). In contrast, at the same time point, HDC^−/−^ mice exhibited slower leukocyte infiltration and the persistent presence of dead cardiomyocytes in the infarcted zone and delayed replacement with granulation tissue, suggesting defective phagocytosis of injured cells (Fig. 5f). In addition, the results of Masson’s staining showed that the extent of fibrosis slightly increased in the infarcted heart of HDC^−/−^ mice compared to WT mice 7 days after MI, though this difference did not reach significance (see Supplementary Fig.S1). Taken together, these data suggest that histamine modulates the infiltration and differentiation of macrophages from IMCs in the infarcted heart, which may contribute to the protective role of histamine during AMI.

### Histamine relieves myocardial injury in MI through Histamine-1 (H_1_R) and Histamine-2 (H_2_R) receptor-dependent signals

**Figure 6:**
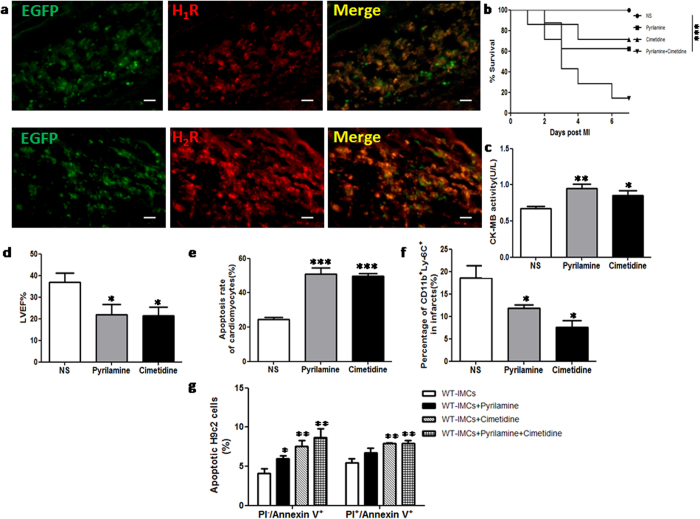
Histamine ameliorates myocardial injury in MI through H_1_ and H_2_ receptors related pathway. (**a**) Immunofluorescence staining showed the expression of H_1_ and H_2_ receptors in the infarcted heart of HDC-EGFP^+^ mice (scale bar = 20 μm). (**b)** WT mice were divided into 4 groups, and were treated with NS (normal saline), pyrilamine (H_1_ receptor antagonist), cimetidine (H_2_ receptor antagonist), and pyrilamine+cimetidine, respectively. At 7d post MI, the survival rate of mice treated with pyrilamine or cimetidine tend to be lower compared to those with NS (NS: 100% vs pyrilamine: 62.5%, *p *= 0.06; NS vs cimetidine: 71.4%, *p *= 0.12; n = 7–8). For those treated with pyrilamine and cimetidine simultaneously, only 1 out of 7 survived 7 days after MI (survival rate: pyrilamine+cimetidine 14.3% versus NS, ***p < 0.001; n = 7–8). (**c)** 1d post MI, the administering of pyrilamine and cimetidine both increased CK-MB activity in the serum of MI mice (**p < 0.01 vs NS, *p < 0.05 vs NS; n = 5–7). (**d)** 7d post MI, the administering of pyrilamine and cimetidine decreased cardiac LVEF (*p < 0.05 vs NS; n = 6). (**e**) The administering of pyrilamine and cimetidine both increased the apoptosis of cardiomyocytes (***p < 0.001 vs NS; n = 4). (**f**) The administering of pyrilamine and cimetidine both suppressed the infiltration of macrophages in the infarcts 1d post MI (*p < 0.05 vs NS; n = 5). (**g**) FACS analysis showing the apoptosis of H9c2 cells co-cultured with bone marrow derived IMCs. The protection of IMCs derived histamine could be abrogated by the antagonists of histamine receptors (**p < 0.01 vs WT, *p < 0.05 vs WT; n = 4).

To clarify which histamine receptor was responsible for the effect of histamine, we first performed immunofluorescence staining with anti-H_1_R and anti-H_2_R and confirmed that expression of H_1_ and H_2_ receptors could be detected on both cardiomyocytes and IMCs, with particularly high expression in IMCs (Fig. 6a). Furthermore, we pre-treated WT mice with pyrilamine (H_1_ receptor antagonist) and cimetidine (H_2_ receptor antagonist), respectively. The 7-day survival rate of MI mice treated with pyrilamine or cimetidine tended to be lower than MI mice treated with normal saline (Fig. 6b), although these differences did not reach significance (*p *= 0.06, *p *= 0.12, respectively). For those animals treated with pyrilamine and cimetidine simultaneously, only 1 out of 7 mice survived 7 days after MI(*p *< 0.001, Fig. 6b). The results of CK-MB activity at 1 day and LVEF value at 7 days also showed the same trend as the overall survival rate (Fig. 6c,d). The results of TUNEL assay confirmed that pyrilamine and cimetidine could both accelerate the apoptosis of α-actinin^+^ cardiomyocytes (Fig. 6e). In addition, at 1 day after MI, FACS data demonstrated that both pyrilamine and cimetidine could suppress the infiltration of macrophages (Fig. 6f). The results of *in vitro* studies using H9c2 cells co-cultured with IMCs showed that treatment with pyrilamine and cimetidine repressed the anti-apoptotic effect of IMCs-derived histamine on H9c2 cells, confirming a role for H_1_ and H_2_ receptors (Fig. 6g). We also administered histamine receptors antagonists plus exogenous histamine in HDC^−/−^ mice, and confirmed the protective role of histamine in the infarcted hearts via H_1_R and H_2_R dependent signaling pathways (see Supplementary Fig.S2). Taken together, these data demonstrate that histamine extenuates myocardial injury during MI through both H_1_ and H_2_ receptor dependent signaling pathways.

## Discussion

In the present study, a sustained elevation of histamine has been documented in the serum of both patients and mice after acute myocardial infarction (AMI). AMI may trigger the egress of histamine-producing HDC^+^ immature myeloid cells (IMCs) from the bone marrow and spleen into the circulation. Genetic deficiency of histamine production exacerbates the injury of the infarcted heart, which may be attributed to the repression of macrophage infiltration and promotion of cardiomyocyte apoptosis.

Our finding of an elevation in serum histamine after AMI is consistent with previous studies^8^,^9^,^10^,^12^. The degranulation of mast cells^16^,^17^,^18^ and activation of sympathetic nerves^30^ were reported to be responsible for the immediate elevation of histamine after the onset of AMI, but mast cells are able to provide only a temporary pulse of histamine release. In a previous study using HDC-EGFP transgenic reporter mice^26^, CD11b^+^Gr-1^+^ IMCs were identified as the major HDC-expressing tissue in inflammation associated tumorigenesis. This suggests that HDC^+^ IMCs could be the persistent and unrecognized cellular source of endogenous histamine in the process of AMI. As expected, a large number of HDC-EGFP expressing CD11b^+^Gr-1^+^ IMCs were detected in the infarcted heart and peripheral blood. Immediately after the onset of MI (e.g. day 1), numerous HDC^+^ IMCs are released into the peripheral blood from the spleen and bone marrow and infiltrate into the infarcted myocardium, which was followed by their expansion in the spleen and bone marrow reservoirs at later stages (day 3 to day 7 post MI). The results from previous studies and ours clarified the major origin of histamine-secreting cells in MI.

Sufficient recruitment of leukocytes, especially macrophages, is necessary for an effective healing process in the early phase of AMI^27^,^28^,^29^. Massive infiltration of macrophages allows phagocytosis of dead cadiomyocytes, facilitates the formation of granulation tissue, and rapidly confines the injury. At the earliest phase of AMI (first 24 hours), we observed significantly decreased macrophages in the infarcts in histamine deficiency mice compared to WT mice. The mechanisms for the protective effect of histamine might include altered permeability of microvessels and adhesion molecule expression^31^,^32^,^33^,^34^. Insufficient macrophages infiltration in histamine deficiency mice leads to delayed replacement of dead cardiomyocytes, which might contribute to prolonged recruitment of macrophages and inflammation. As we have shown, the total numbers of macrophages (CD11b^+^Ly6C^+^) and the number of M1-type macrophages (CD11b^+^Ly6C^high^) continued to increase over time in HDC^−/−^ mice, and were nearly 2-fold higher in HDC^−/−^ mice compared to WT counterparts at 7 days post MI. As suggested by earlier studies, too many redundant macrophages, especially CD11b^+^Ly6C^high^ M1-type macrophages, might be harmful to tissue repair in the later phase of MI^35^,^36^.

The role of histamine in apoptosis has been investigated in the cardiovascular system^10^,^13^,^24^,^37^ and other systems^38^,^39^,^40^, yet the results are still controversial. The different concentrations of exogenous histamine administered in these studies may lead to variable effects on cell apoptosis. In the present study, we established a co-culture system with H9c2 cells and IMCs sorted from the bone marrow of HDC^−/−^ and WT mice. FACS data demonstrated that histamine deficiency could increase the apoptosis of H9c2 cells induced by hydrogen peroxide treatment, which could be abrogated by exogenous histamine (5*10^−5 ^M). Furthermore, the evidence from our studies suggest that both H_1_ and H_2_ receptors are likely responsible for such an effect. This anti-apoptotic effect is acting through the functions of IMCs, for administering histamine (higher than 10^−5 ^M) alone could increase the apoptosis of H9c2 cells. Given that histamine could promote macrophage differentiation from IMCs^26^, we propose that such functions of IMCs and macrophages may include the debridement of injured cardiomyocytes^41^,^42^ and release of some anti-apoptotic cytokines, such as VEGF, TGF-β and hepatocyte growth factor (HGF)^41^,^42^,^43^,^44^,^45^. However, the current study also showed that excessive exogenous histamine (4 mg/kg/d) could be detrimental to WT mice subjected to MI (see Supplementary Fig.S3). Thus, we believe the physiological level of histamine is most favorable for the healing process of MI. Future studies will focus on identifying the intracellular signaling pathways induced by histamine, which mitigate cardiomyocyte apoptosis in the setting of oxidative stress.

Some clinical studies have suggested that AMI patients with higher serum IgE levels had a better prognosis, which may be attributed to increased histamine release from mast cells^46^,^47^. On the other hand, several studies concerning chronic heart failure (CHF) patients indicated that histamine receptors antagonists, especially H_2_ receptor antagonists, could improve cardiac function^48^,^49^,^50^. Based on these reports and our own findings, we propose that histamine may have variable roles in different stages of MI. In the early stages of MI, histamine promotes the infiltration of macrophages and facilitates the debridment of dead cardiomyocytes and rapidly confines the injury. In the later stages, the role of histamine might be involved in the cardiac remodeling and fibrogenesis. In a recent study, excessive remodeling mediated by H_2_ receptor activation has been demonstrated in a transverse aortic constriction (TAC) model^24^. Given a variety of histamine receptors (H_1_R, H_2_R) expressed on the cardiomyocytes, fibroblast, endothelial cells, and immune cells, further investigations are needed to better understand the role of histamine and histamine receptors in cardiac diseases.

In conclusion, HDC-expressing IMCs are the principle cellular source of histamine in AMI, and endogenous histamine plays a protective role against cardiomyocyte apoptosis and cardiac dysfunction in AMI. Thus, H_1_ receptor or H_2_ receptor antagonists should be used cautiously in the early stages of AMI, until further clinical and basic studies are completed.

## Methods

### Patients’ characteristics and serum histamine determination

Serum was taken from patients who were admitted to our hospital (Zhongshan Hospital, Fudan University). All of these patients claimed to have symptoms, including chest pain, chest tightness, palpitation, etc. Angiography was conducted to determine the diagnosis of acute myocardial infarction (AMI). 14 patients with confirmed AMI (2.5 hours to 15 hours from onset) and 10 patients with coronary artery diseases (not MI) were selected. Serum concentration of histamine was determined using Histamine ELISA Kit (EA213/96, Eagle Biosciences), and detected by a microplate reader (SpectraMax M5). The investigations complied with the ethical guidelines of the 1975 Declaration of Helsinki and were approved by the review boards on human subject research in our institution (Zhongshan Hospital, Fudan University). Informed consent was obtained from all participants.

### Animal models

HDC-EGFP and HDC knockout (HDC^−/−^) mice were generously provided by Professor Timothy C. Wang from Columbia University. The generation of HDC-EGFP and HDC^−/−^ mice has been described in previous papers^26^,^51^. Balb/C mice and C57BL/6 mice were purchased from the Department of Laboratory Animal Science, Fudan University, to serve as background controls. All mice were housed under specific-pathogen-free conditions in an animal room with a 12/12 hr day/night cycle with free access to water and food. This study was performed in strict accordance with the recommendations from the Guide for Animal Management Rules from the Ministry of Health of the People’s Republic of China. The protocol was approved by the Committee on the Ethics of Animal Experiments of Fudan University (approval reference number: SY2014.2.001.002). Anesthesia was performed by inhalation of 1.0–2.0% isoflurane gas. Perioperative mice received piritramide (10 mg/kg body weight) and postoperative tramadolhydrochloride (2.5 mg/100 ml drinking water) for the first seven days after operation. The physical condition of the animals was evaluated two times per day.

### Myocardial infarction model

Surgery to induce myocardial infarction was performed in the mice as described previously^52^. In brief, the mice were anesthetized by inhalation of isoflurane, were intubated with a 22-G intravenous catheter, and then were fully anesthetized with 1.0–2.0% isoflurane gas while being mechanically ventilated on a positive pressure ventilator. Left thoracotomy was performed at the fourth intercostal space, and the pericardium was stripped to expose the heart. The left descending coronary artery was identified and occluded with an 8–0 silk ligature that was placed around it. The success of the ligation was confirmed when the anterior wall of the left ventricle turned pale. The chest cavity was closed, and the animal was placed in a cage on a heating pad. Sham-operated mice underwent the same surgical procedures except that the suture placed under the left anterior descending artery was not tied. From 3 days before surgery, histamine (4 mg/kg/d), H_1_R antagonist (pyrilamine, 10 mg/kg/d) and H_2_R antagonist (cimetidine, 10 mg/kg/d) were administered intraperitoneally daily until euthanasia.

### Quantitative reverse transcription-PCR (qRT-PCR)

Quantitative RT-PCR for HDC mRNA was done as described on an Applied Biosystems AB 7500 Real Time PCR system. Total RNA in the blood cells of mice was extracted using QIAamp RNA Blood Mini Kit according to manufacturer’s instructions. After DNase I (Takara) treatment, RNA was reverse transcribed using Prime Script 1st Strand cDNA Synthesis Kit (Takara). PCR reactions were prepared using SYBR Premix Ex TaqII (TaKaRa), followed by quantitative PCR on an Applied Biosystems AB 7500 Real Time PCR system. The results were standardized to control values of glyceraldehyde 3-phosphate dehydrogenase (GAPDH). HDC forward primer: 5′-TTAGTCTTTGGGTGTTCCTGGTCA-3′; reverse: 5′-CCC TGTTGCTTGTCTTCCTCAATA-3′. GAPDH forward primer: 5′-GACATCAAGAAGGTGGTGAAGCAG-3′; reverse: 5′-ATACCAGGAAATGAGCTTGACAAA-3′.

### Echocardiography

Transthoracic echocardiography was performed using the Vevo770 imaging system (VisualSonics, Inc.) 7days after surgery. The mice were anesthetized with 1.0–2.0% isoflurane and placed on a heating pad to maintain their body temperatures. The left ventricular dimensions were quantified by digitally recorded two-dimensional short-axis M-mode tracings at the level of the papillary muscles, to allow for consistent measurement at the same anatomic location indifferent mice; at least three consecutive beats were evaluated. Left ventricular (LV) wall thickness and internal dimensions were measured. LV ejection fraction(LVEF) and LV fractional shortening (LVFS) were calculated.

### Flow cytometry analysis

Mice were sacrificed at 1 day and 7 days after MI. The blood, spleen, bone marrow and infarcted myocardium were collected and made into single-cell suspensions for flow cytometry, as previously described^26^,^52^. The cells were stained with a mixture of antibodies (anti-CD11b-APC, anti-Gr-1-PerCP-Cy5.5, anti-Ly6C-PE; BD Biosciences). Data were acquired using an LSRII flow cytometer (BD Biosciences) and were analyzed with FlowJo7 software (Tree Star, Inc.).

### Histology, Immunofluorescence, Immunohistochemistry and TUNEL assay

Hearts from HDC-EGFP mice were fixed with 4% paraformaldehyde for 24 hrs followed by 30% sucrose overnight for frozen tissue. Primary antibodies to CD11b (Abcam), Gr-1(BD Biosciences), H_1_R (Novus Biologicals) and H_2_R (Novus Biologicals) were used for immunofluorescence on frozen sections. Secondary antibodies included Texas Red-conjugated rabbit-specific antibody (Abcam) and PE–conjugated rat-specific antibody (Abcam). Hearts from WT and HDC^−/−^ mice were fixed with 10% formalin for paraffin embeddings. Hematoxylin-eosin staining was performed. Primary antibody to CD68 (Abcam) and Dako REAL^TM^ Envision^TM^ Detection System (Peroxidase/DAB+) were used for immunohistochemistry to detect macrophages. Fibrosis was analyzed by Masson staining. Apoptosis was evaluated via the terminal deoxynucleotidyl transferase-mediated dUTP nick-end labeling (TUNEL) method on paraffin sections, using the *In Situ* Cell Death Detection Kit (Roche) according to the protocol provided by the manufacturer. Cardiomyocytes were stained using anti-α-actinin antibody. It’s a frequently-used marker of cardiomyocytes^53^. The apoptosis index of cardiomyocytes was determined by counting TUNEL-positive nuclei in 15 different fields per section, and it is expressed as a percentage of the total nuclei of cardiomyocytes.

### Measurement of CK-MB activity and cytokine abundance by ELISA

Serum from mice sacrificed at 1 day after MI was used for the measurement of CK-MB activity using an ELISA Kit (R&D System). The expressions of IL-6 and IL-1 at 1 day after MI were measured with respective ELISA Kits (R&D System).

### Infarct size assessment

After 24 hrs of ischemia, the infarct size was determined with triphenyltetrazolium chloride (TTC) staining. Briefly, the mice were sacrificed and the hearts were removed and sliced into five 1.0-mm thick sections perpendicular to the long axis. The sections were then incubated with 1% TTC (Sigma) in phosphate solution at 37 °C for 10 min. The areas of infarcted tissue (TTC-negative staining area) and the whole left ventricle were determined by computer morphometry using Image-Pro Plus 6.0 software.

### Cell culture and apoptosis assay

Bone marrow from Balb/C and HDC^−/−^mice was collected and made into single-cell suspensions as previously described^26^,^52^. CD11b^+^IMCs were sorted by magnetic beads. IMCs (approximately 1.5 million/well) and H9c2 cells were co-cultured in six-well plates with 10% FBS DMEM medium. H_2_O_2_ (100 μmol/L) was added to provide oxidative stress. Histamine (5*10^−5 ^M, 10^−5^ M), H_1_R antagonist (1 μM pyrilamine) and H_2_R antagonist (50 μM cimetidine) were added to the medium. Mixed cells were harvested 48 hrs later, and Annexin V-PI staining was conducted to detect apoptosis by flow cytometry. Cell size and antibody to CD11b were used to distinguish H9c2 from IMCs.

### Statistical analysis

The data are expressed as means ± SEMs. Comparisons between the two groups were assessed by the *t*test. The Chi-square test (or Fisher’s exact test when appropriate) was used to compare discrete variables between different groups. A *P* value less than 0.05 was considered significant.

## Additional Information

**How to cite this article**: Deng, L. *et al.* Histamine deficiency exacerbates myocardial injury in acute myocardial infarction through impaired macrophage infiltration and increased cardiomyocyte apoptosis. *Sci. Rep.* **5**, 13131; doi: 10.1038/srep13131 (2015).

## Supplementary Material

Supplementary Information

## Figures and Tables

**Table 1:** Echocardiographic results at 7-day post MI.

Parameters	WT/Sham(n=6)	HDC^−/−^/Sham(n=5)	WT/MI(n=6)	HDC^−/−^/MI(n=6)	HDC^−/−^+His/MI(n=7)
LVEF(%)	71.09 ± 2.59	68.49 ± 3.82	36.8 ± 4.35^***^	24.5 ± 2.49*	34.61 ± 3.59^**#**^
LVFS(%)	39.89 ± 2.06	37.73 ± 3.03	17.77 ± 2.4^***^	11.24 ± 1.2*	16.52 ± 1.95^**#**^
LVEDd(mm)	3.51 ± 0.11	3.42 ± 0.34	4.36 ± 0.1^***^	4.44 ± 0.2	4.37 ± 0.18
LVESd(mm)	2.22 ± 0.17	2.15 ± 0.26	3.59 ± 0.15^***^	3.95 ± 0.2	3.66 ± 0.21
LVAWd(mm)	0.82 ± 0.11	1.03 ± 0.18	0.73 ± 0.08	0.76 ± 0.11	0.81 ± 0.07
LVAWs(mm)	1.27 ± 0.11	1.4 ± 0.14	0.89 ± 0.15	0.82 ± 0.08	1.02 ± 0.11

His: histamine, 4 mg/kg/d; LVEF: left ventricular ejection fraction; LVFS: left ventricular fraction shortening; LVEDd: left ventricular end-diastolic diameter; LVESd: left ventricular end-systolic diameter; LVAWd: left ventricular diastolic anterior wall thickness; LVAWs: left ventricular systolic anterior wall thickness.

***P < 0.001 vs WT/Sham; *P < 0.05 vs WT/MI; ^#^*P *< 0.05 vs HDC^−/−^/MI.
