# Supplementary material for: Histamine deficiency exacerbates myocardial injury in acute myocardial infarction through impaired macrophage infiltration and increased cardiomyocyte apoptosis
Source: Sci Rep. 2015 Aug 17;5:13131. doi: 10.1038/srep13131 (PMC4642534; doi:10.1038/srep13131)
Supplement: Supplementary Information [file srep13131-s1.pdf]

SUPPLEMENTARY INFORMATION

Histamine deficiency exacerbates myocardial injury in acute myocardial infarction through impaired macrophage infiltration and increased cardiomyocyte apoptosis

Long Deng[1,2#] , Tao Hong[1,2#], Jinyi Lin[1], Suling Ding[1], Zheyong Huang[1], Jinmiao Chen[1,2], Jianguo Jia[1], Yunzeng Zou[1,3], Timothy C. Wang[4], Xiangdong Yang[1*], Junbo Ge[1,3*]

1. Shanghai Institute of Cardiovascular Diseases, Zhongshan Hospital, Fudan University, Shanghai, 200032, China
2. Department of Cardiac Surgery, Zhongshan Hospital, Fudan University, Shanghai, 200032, China
3. Institutes of Biomedical Sciences, Fudan University, Shanghai, China.
4. Department of Medicine and Irving Cancer Research Center, Columbia University, New York, NY 10032, USA.

Supplementary Figures S1-3

a

b

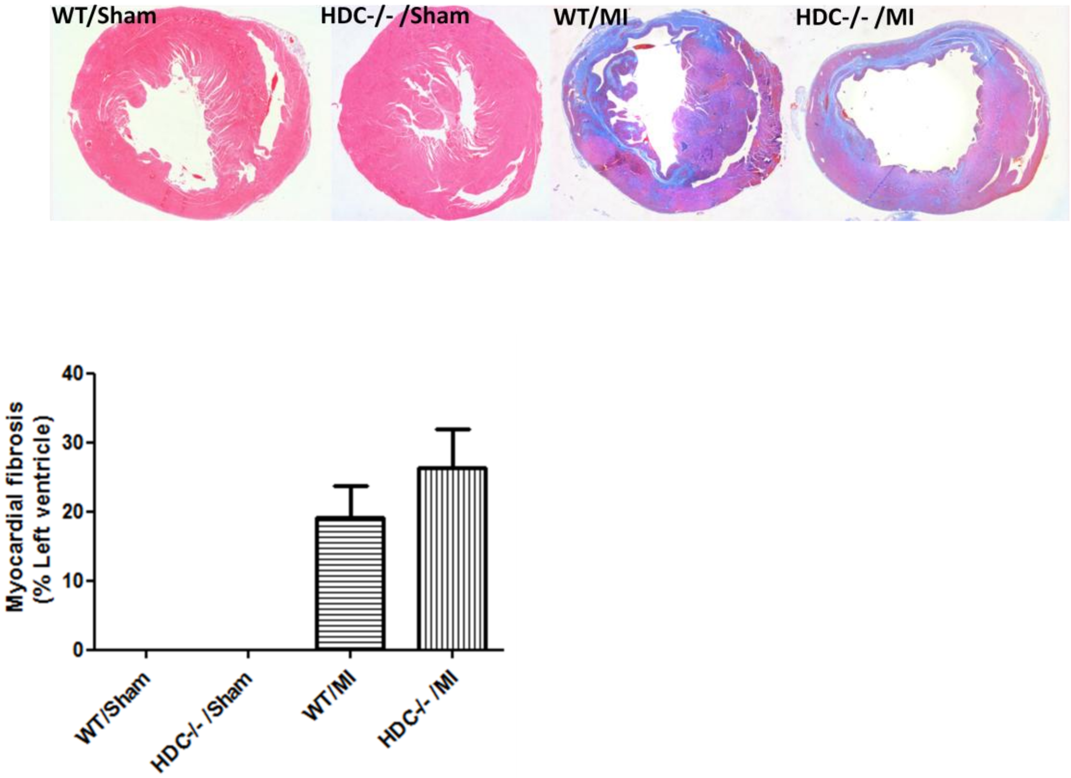

Supplementary Figure S1: The effect of histamine deficiency on myocardial fibrosis following AMI

a, Representative images of Masson staining. **b**, The extent of fibrosis slightly increased in the infarcted heart of $HDC^{-/-}$ mice 7 days after MI, though it did not reach a significant level (*p*=0.37, n=4).

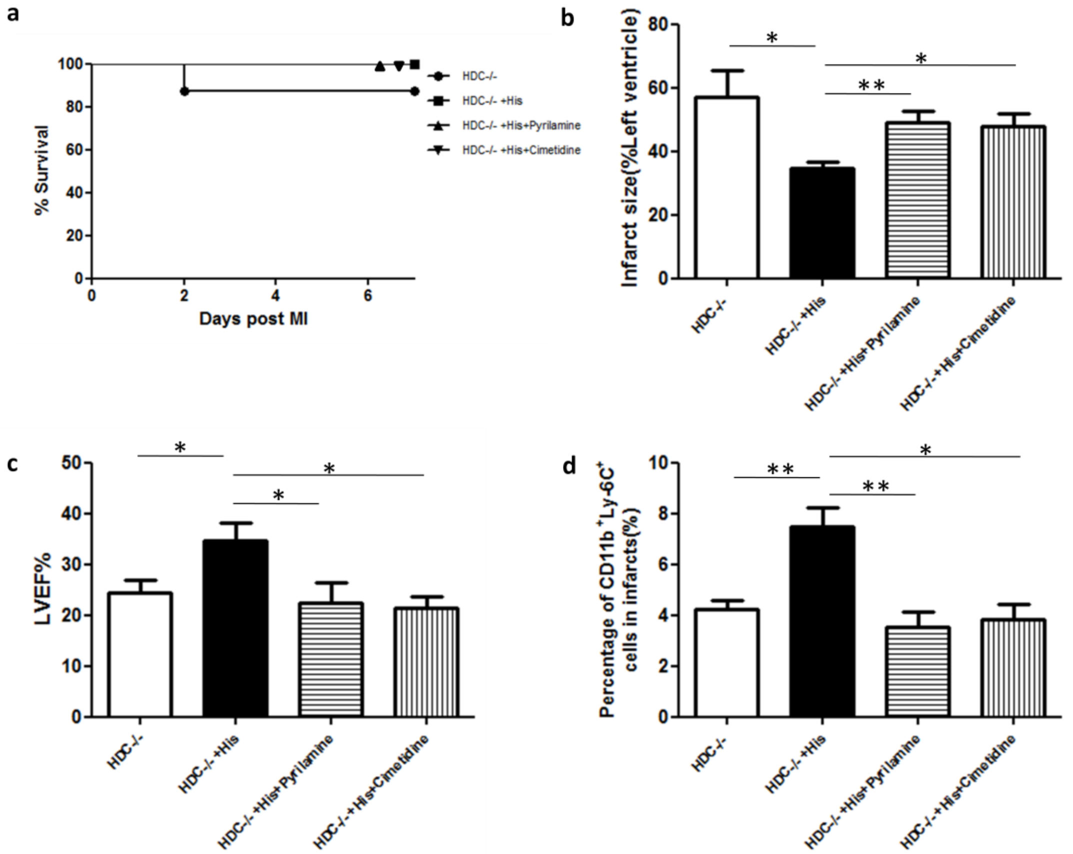

Supplementary Figure S2: The effects of histamine receptors antagonists on HDC$^{-/-}$ mice plus exogenous histamine

a, Histamine receptors antagonists did not compromise the survival of HDC$^{-/-}$ mice plus exogenous histamine. **b**, The infarct size was increased by the administering of pyrilamine and cimetidine (***p*<0.01 vs HDC$^{-/-}$ +His, **p*<0.05 vs HDC$^{-/-}$ +His; n=4). **c**, LVEF% (7d post MI) was decreased by the administering of pyrilamine and cimetidine (**p*<0.05 vs HDC$^{-/-}$ +His; n=6-8). **d**, The administering of pyrilamine and cimetidine both suppressed the infiltration of macrophages in the infarcts 1d post MI (***p*<0.01 vs HDC$^{-/-}$ +His, **p*<0.05 vs HDC$^{-/-}$ +His; n=4).

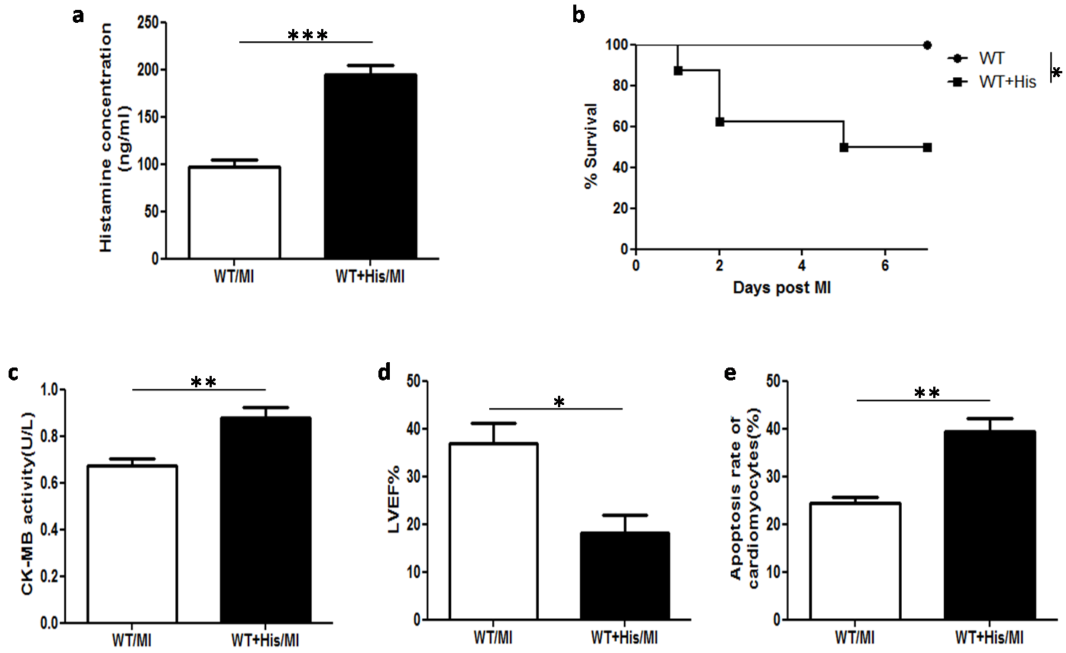

Supplementary Figure S3: Excessive exogenous histamine could be detrimental to the mice subjected to MI

a, Exogenous histamine administration (4mg/kg, intraperitoneal injection) increased the levels of histamine in WT mice significantly (***p<0.001 vs WT; n=5). **b**, Exogenous histamine compromised the survival of WT mice after AMI (*p<0.05 vs WT; n=8). **c**, The level of CK-MB activity was increased by the administering of exogenous histamine (**p<0.01 vs WT; n=5 and 7). **d**, LVEF% (7d post MI) was decreased by the administering of exogenous histamine (*p<0.05 vs WT; n=4 and 6). **e**, The administering of exogenous histamine increased the apoptosis of cardiomyocytes (**p<0.01 vs WT; n=4).
